# Malaria rapid diagnostic test as point-of-care test: study protocol for evaluating the VIKIA® Malaria Ag Pf/Pan

**DOI:** 10.1186/s12936-015-0633-3

**Published:** 2015-03-14

**Authors:** Saorin Kim, Sina Nhem, Dany Dourng, Didier Ménard

**Affiliations:** Institut Pasteur du Cambodge, Phnom Penh, Cambodia

**Keywords:** Malaria, *Plasmodium falciparum*, Fapid diagnostic tests, Point-of-care, Community health workers, Cambodia

## Abstract

**Background:**

Malaria rapid diagnostic tests (RDTs) are generally considered as point-of-care tests. However, most of the studies assessing the performance of malaria RDTs are conducted by research teams that are not representative of the classical end-users, who are typically unskilled in traditional laboratory techniques for diagnosing malaria. To evaluate the performance of a malaria RDT by end-users in a malaria-endemic area, a study protocol was designed and the VIKIA® Malaria Ag Pf/Pan test, previously evaluated in 2013, was re-evaluated by representative end-users.

**Methods:**

Twenty end-users with four different profiles in seven communes in Kampot Province (Cambodia) were selected. A set of 20 calibrated aliquots, including negative samples, low positive samples (200 parasites/μL of *Plasmodium falciparum* and *Plasmodium vivax*) and high positive samples (2,000 parasites/μL of *P. falciparum* and *P. vivax*) was used. Testing was performed directly by the end-users without any practical training on the VIKIA® Malaria Ag Pf/Pan kit.

**Results:**

All results obtained by the end-users were consistent with the expected results, except for the low positive (200 parasites/μL) *P. vivax* aliquot (35% of concordant results). No significant difference was observed between the different end-users. End-user interviews evaluating ease-of-use and ease-of-reading of the VIKIA® Malaria Ag Pf/Pan kit recorded 159 positive answers and only one negative answer. Out of 20 end-users, only one considered the test was not easy to perform with the support of the quick guide.

**Conclusions:**

The data presented in this study clearly demonstrate that the performance of the VIKIA® Malaria Ag Pf/Pan test when performed by traditional end-users in field conditions is similar to that obtained by a research team and that this RDT can be considered as a point-of-care tool/assay. Furthermore, the protocol designed for this study could be used systematically in parallel to conventional evaluation studies to determine the performance of malaria RDTs in field conditions.

**Electronic supplementary material:**

The online version of this article (doi:10.1186/s12936-015-0633-3) contains supplementary material, which is available to authorized users.

## Background

Malaria remains one of the most important infectious diseases in tropical area, affecting over 300 million people every year, mainly in sub-Saharan Africa [1,2]. As recommended by the World Health Organization (WHO), the management of suspected malaria cases relies on early diagnosis and prompt and effective treatment based on artemisinin-combined therapy (ACT) [3].

Malaria diagnosis has long been based on the microscopy examination of Giemsa-stained blood film, despite the requirement for highly qualified microscopists and reliable equipment, which are often lacking in remote areas where malaria is most prevalent [4]. For over a decade, the development of malaria rapid diagnostic tests (RDTs) has enabled reliable biological diagnostic testing in all situations where previously only clinical diagnosis was available [5]. The main advantages of the malaria RDTs are that they are easy to use, do not require electricity or complex equipment and the results are available in 15–30 minutes after finger-prick blood collection [6]. These lateral flow immunochromatographic tests are usually presented in various formats (dipstick, plastic cassette or card). They contain antibodies conjugated to colloidal gold or latex particles, which bind specifically with parasite antigens. They are generally based on the detection of the histidine-rich-2 protein (HRP-2) specific to *Plasmodium falciparum* but often combined with the detection of other antigens common to all species, such as lactate dehydrogenase or aldolase. These combined RDTs can detect both *P. falciparum* and non-*P. falciparum* (*Plasmodium vivax, Plasmodium ovale* and *Plasmodium malariae*) infections [7].

Over the past few years, the number of malaria RDTs and the scale of their use have rapidly increased. With over 100 commercially produced RDTs, this market has led to a proliferation in product availability against a background of relatively low capacity for regulation and quality control in many low-resourced, malaria-endemic countries. The variable quality of malaria RDTs, and consequently their diagnostic performance, have made it difficult for policy makers to determine which tests are the most suitable [5], even with the implementation of the lot-testing programme, led by WHO in partnership with the UNICEF/UNDP/World Bank/WHO Special Programme for Research and Training in Tropical Diseases (TDR) and the Foundation for Innovative New Diagnostics (FIND) [8].

At present, malaria RDTs are considered as point-of-care tests because they are mainly used by health care volunteers at community level, in remote malaria areas. Their use in field conditions allows an early diagnosis of malaria in any febrile patient and prompt treatment of confirmed cases. However, most of the studies aiming to assess the performance of malaria RDTs are conducted by research teams that are not representative of the classical end-users, who are typically unskilled in traditional laboratory techniques for diagnosing malaria. Consequently, only the ‘intrinsic’ performance of malaria RDTs is evaluated and information regarding their ‘global’ performance (including ease of use, execution, interpretation of results, and adherence to test results) when they are used by the traditional end-users is lacking.

In this context, a protocol for evaluating the global performance of malaria RDTs by end-users in malaria-endemic areas was developed. As an example, the performance of the VIKIA® Malaria Ag Pf/Pan test, previously assessed in 2013 [9], was re-evaluated by representative end-users, using well characterized and calibrated blood samples in several testing sites in Cambodia. Results obtained by the end-users were compared to the corresponding expected results. In addition, a questionnaire assessing the ease-of-use of the RDT with the support of the package insert and the quick guide was also developed.

## Methods

### VIKIA® Malaria Ag Pf/Pan test

The VIKIA® Malaria Ag Pf/Pan (IMACCESS/bioMérieux, Lyon, France) is a test based on immunochromatographic technology for detecting *P. falciparum* and other species of *Plasmodium* (*P. vivax, P. malariae* and *P. ovale*). This test is a ready-to-use cassette device, requiring the operator to transfer 5 μL of blood in the sample well with an appropriate device supplied in the kit, add five drops of the lysis buffer in the buffer well and read the results visually after 20 minutes (see Additional files 1 and 2; SF1 Quick guide for using the VIKIA® Malaria Ag Pf/Pan RDT and SF2 Package Insert of the VIKIA® Malaria Ag Pf/Pan RDT).

### Participating communes and end-users

**Figure 1:**
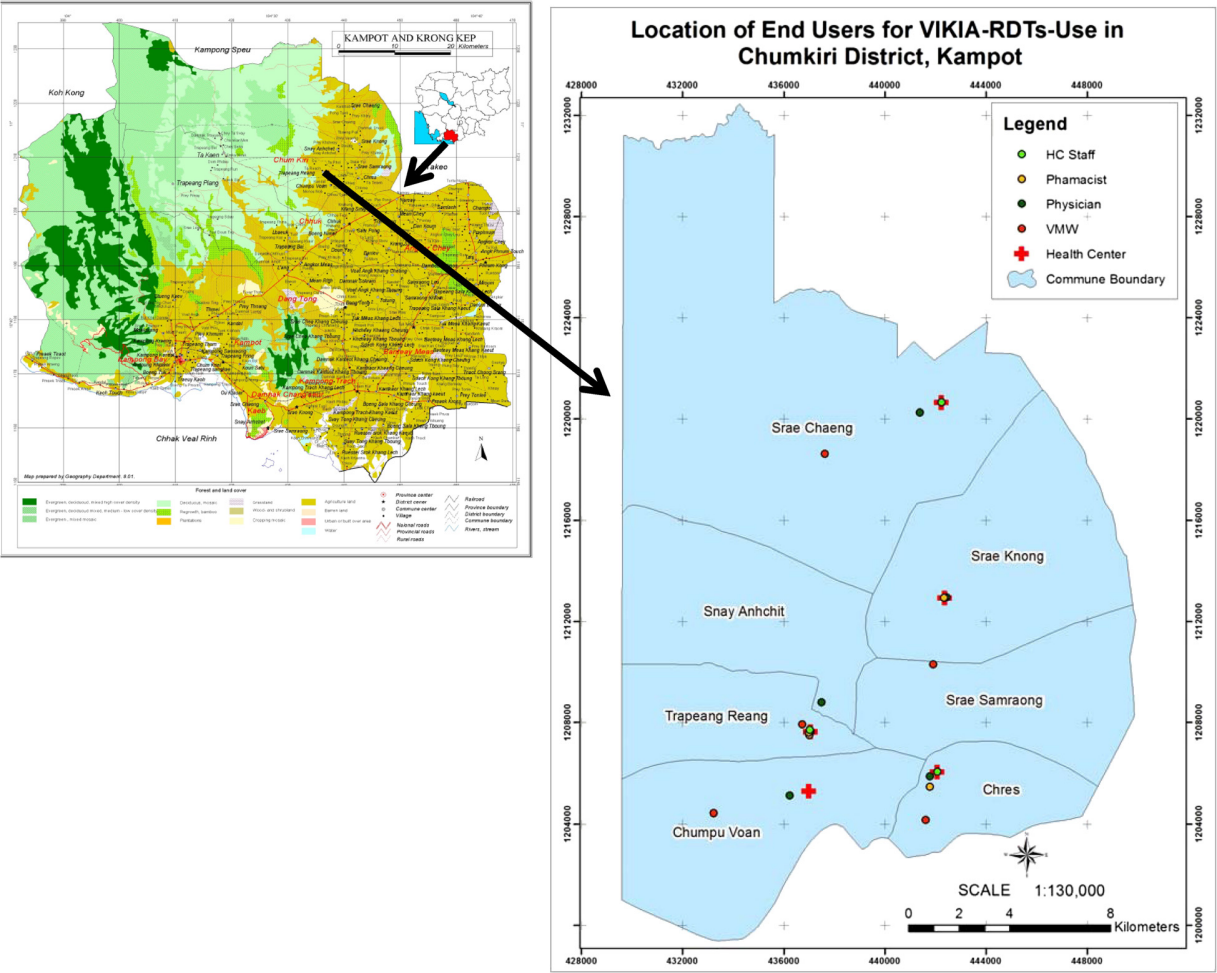
**Location of the selected end-users, Kampot Province, Cambodia.**

Seven communes in Kampot Province (Cambodia) were selected for this multisite, blinded study. Four different end-user profiles were considered as representative of the typical malaria RDTs end-users found in malaria-endemic areas: health medical centre staff, private physician, community health worker (village malaria worker (VMW)) and private pharmacist. Five individuals of each end-user profiles were enrolled in the study, as presented in Figure 1 and Table 1.

**Table 1:** **List of selected end-users, Kampot Province, Cambodia**

**End-user**		**Location**			
		**Village**	**Commune**	**District**	**Province**
#1	Health medical centre staff	TrapaingVeng	TrapaingRaing	Chumkiri	Kampot
#2	Pharmacist	TrapaingVeng	TrapaingRaing	Chumkiri	Kampot
#3	Village malaria worker	TrapaingVeng	TrapaingRaing	Chumkiri	Kampot
#4	Physician	Taphoul	Snay Anchit	Chumkiri	Kampot
#5	Pharmacist	TrapaingVeng	TrapaingRaing	Chumkiri	Kampot
#6	Health medical centre staff	Rokarthmey	Srae Samraong	Chumkiri	Kampot
#7	Physician	Kamnob	Srae Chaeng	Chumkiri	Kampot
#8	Village malaria worker	PangTeuk	Srae Chaeng	Chumkiri	Kampot
#9	Pharmacist	TrapaingVeng	TrapaingRaing	Chumkiri	Kampot
#10	Physician	Mononob	Chumpu Voan	Chumkiri	Kampot
#11	Health medical centre staff	TrapaingVeng	TrapaingRaing	Chumkiri	Kampot
#12	Village malaria worker	Mononob	Chumpu Voan	Chumkiri	Kampot
#13	Physician	Chres	Chres	Chumkiri	Kampot
#14	Village malaria worker	Thmear	Chres	Chumkiri	Kampot
#15	Pharmacist	Chres	Chres	Chumkiri	Kampot
#16	Health medical centre staff	TrapaingVeng	TrapaingRaing	Chumkiri	Kampot
#17	Health medical centre staff	PreyYao	Srae Khnong	Chumkiri	Kampot
#18	Physician	PreyYao	Srae Khnong	Chumkiri	Kampot
#19	Pharmacist	PreyYao	Srae Khnong	Chumkiri	Kampot
#20	Village malaria worker	PreyKhmao	Srae Khnong	Chumkiri	Kampot

### Sample characteristics

Three types of sample (negative, low positive, high positive) were tested in replicates of four, corresponding to a panel of 20 aliquots, as followed: one negative sample, one low positive sample at 200 parasites of *P. falciparum*/μL, one low positive sample at 200 parasites of *P. vivax*/μL, one high positive sample at 2,000 parasites of *P. falciparum*/μL and one high positive sample at 2,000 parasites of *P. vivax*/μL.

The set of 20 coded aliquots was prepared according to the Standard Operating Procedures (SOP) developed in the Methods Manual for Laboratory QC Testing of Malaria RDTs (Version 7, June 2014) and codified as described in Table 2. Briefly, the preparation of positive parasite samples was performed in three steps: i) blood sample collection from consenting malaria patients (strong positive detection by RDT and parasite density > 2,000 parasites/μL of blood by microscopy); ii) collection of parasite-free (controlled by PCR [10]) and virus-free (controlled by serological screening for HIV, hepatitis B and hepatitis C infections) blood from the Phnom Penh blood bank and, iii) dilution of the parasite-positive bloods in low (200 parasites/μL) and high (2,000 parasites/μL) parasite densities and preparation of parasite-negative QC samples and aliquoting (50 μL) in cryotubes frozen at −80°C.

**Table 2:** **List of the set of 20 parasite-calibrated blood aliquots used by the selected end-users, Kampot Province, Cambodia**

**Aliquot ID**	***Plasmodium*** **species**	**Parasite density (/μL)**
Aliquot 1	*P. vivax*	2,000
Aliquot 2	*P. vivax*	200
Aliquot 3	-	
Aliquot 4	*P. vivax*	200
Aliquot 5	*P. falciparum*	200
Aliquot 6	*P. falciparum*	2,000
Aliquot 7	*P. vivax*	2,000
Aliquot 8	-	
Aliquot 9	*P. falciparum*	200
Aliquot 10	*P. vivax*	2,000
Aliquot 11	*P. falciparum*	2,000
Aliquot 12	*P. falciparum*	200
Aliquot 13	*P. falciparum*	2,000
Aliquot 14	*P. vivax*	2,000
Aliquot 15	*P. vivax*	200
Aliquot 16	*P. falciparum*	2,000
Aliquot 17	-	
Aliquot 18	-	
Aliquot 19	*P. vivax*	200
Aliquot 20	*P. falciparum*	200

**Figure 2:**
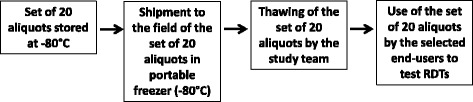
**Flow chart of the use in field conditions of well-characterized and calibrated blood samples (set of 20 aliquots) by selected end-users, Kampot Province, Cambodia.**

Frozen calibrated blood aliquots were transported at −80°C in a portable freezer running on 12 V (works with a cigarette lighter in a car). Defrosting was performed by the research team just before its use by the end-user (Figure 2).

### Training

Before the start of the study, the research team explained the study objective to all end-users. Testing was performed directly by the end-users without any practical training on the VIKIA® Malaria Ag Pf/Pan kit. The only instructions for use were available within the quick guide and the package insert provided in the VIKIA® Malaria Ag Pf/Pan kit (see Additional files 1 and 2).

### Quality control assessment of the calibrated aliquots

The research team performed a quality control assessment of the set of the 20 aliquots at three different times (at the start, middle and end of the study), to confirm the good quality of the aliquots distributed to the users and to verify that no degradation occurred during transport or storage that could affect the expected results.

### Statistical analysis

Data were recorded and analysed using Excel software and MedCalc (MedCalc Software, Belgium). The Chi-squared test was used to compare the proportion of positive results among end-users. A P-value <0.05 was considered to indicate statistical significance.

## Results

### Quality assessment testing results

Three successive quality assessments were performed by the research team: firstly in Phnom Penh at Institut Pasteur in Cambodia before going into the field, secondly and thirdly, seven and 14 days later, respectively, in the field. Results are presented in Table 3.

**Table 3:** **Results of the three quality assessments performed by the research team**

**Aliquot ID**		**QA 1 (Day 0)**				**QA 2 (Day 7)**				**QA 3 (Day 14)**			
		**RDT reading results**			**Interpretation**	**RDT reading results**			**Interpretation**	**RDT reading results**			**Interpretation**
		**C**	***Pf***	**Pan**		**C**	***Pf***	**Pan**		**C**	***Pf***	**Pan**	
3	Negative	P	A	A	**Concordant**	P	A	A	**Concordant**	**P**	A	A	**Concordant**
8	Negative	P	A	A	**Concordant**	P	A	A	**Concordant**	**P**	A	A	**Concordant**
17	Negative	P	A	A	**Concordant**	P	A	A	**Concordant**	**P**	A	A	**Concordant**
18	Negative	P	A	A	**Concordant**	P	A	A	**Concordant**	**P**	A	A	**Concordant**
5	*P. falciparum* - 200	P	P	A	**Concordant**	P	P	A	**Concordant**	**P**	**P**	A	**Concordant**
9	*P. falciparum* - 200	P	P	A	**Concordant**	P	P	A	**Concordant**	**P**	**P**	A	**Concordant**
12	*P. falciparum* - 200	P	P	A	**Concordant**	P	P	A	**Concordant**	**P**	**P**	A	**Concordant**
20	*P. falciparum* - 200	P	P	A	**Concordant**	P	P	A	**Concordant**	**P**	**P**	A	**Concordant**
6	*P. falciparum* – 2,000	P	P	P	**Concordant**	P	P	P	**Concordant**	**P**	**P**	**P**	**Concordant**
11	*P. falciparum* – 2,000	P	P	P	**Concordant**	P	P	P	**Concordant**	**P**	**P**	**P**	**Concordant**
13	*P. falciparum* – 2,000	P	P	P	**Concordant**	P	P	P	**Concordant**	**P**	**P**	**P**	**Concordant**
16	*P. falciparum* – 2,000	P	P	P	**Concordant**	P	P	P	**Concordant**	**P**	**P**	**P**	**Concordant**
2	*P. vivax* - 200	P	A	Pw	**Concordant**	P	A	Pw	**Concordant**	**P**	A	Pw	**Concordant**
4	*P. vivax* - 200	P	A	Pw	**Concordant**	P	A	A	*Discordant*	**P**	A	A	*Discordant*
15	*P. vivax* - 200	P	A	Pw	**Concordant**	P	A	Pw	**Concordant**	**P**	A	A	*Discordant*
19	*P. vivax* - 200	P	A	Pw	**Concordant**	P	A	A	*Discordant*	**P**	A	A	*Discordant*
1	*P. vivax* – 2,000	P	A	P	**Concordant**	P	A	P	**Concordant**	**P**	A	**P**	**Concordant**
7	*P. vivax* – 2,000	P	A	P	**Concordant**	P	A	P	**Concordant**	**P**	A	**P**	**Concordant**
10	*P. vivax* – 2,000	P	A	P	**Concordant**	P	A	P	**Concordant**	**P**	A	**P**	**Concordant**
14	*P. vivax* – 2,000	P	A	P	**Concordant**	P	A	P	**Concordant**	**P**	A	**P**	**Concordant**

QA: quality assessment; C: control line; Pf: P. falciparum line (HRP-2 line detection); Pan: Plasmodium line (aldolase detection line); P: positive; A: negative; Pw: positive weak.

Quality assessments 2 and 3 showed a significant degradation of the quality of three out of four aliquots containing *P. vivax* at 200 parasites/μL. For this reason, aliquots ID 4, ID15 and ID19 were excluded from the final analysis of the end-user testing results.

### End-user testing results

Among the 20 selected end-users, the results observed were consistent with the expected results, except for the low positive (200 parasites/μL) *P. vivax* aliquot with 35% of concordant results (Table 4).

**Table 4:** **Results of the tests performed by selected end-users**

**Aliquot status**	**Aliquots used**	**End-users**	**No. of tests performed**	**No. of aliquots declared as**			**% of concordant results**
				**Negative**	***P. falciparum***	**Non-** ***P. falciparum***	
Negative	ID3, ID8, ID17, ID18	Health centre staff	20	20	0	0	100%
		Pharmacist	20	20	0	0	100%
		Village malaria worker	20	20	0	0	100%
		Physician	20	20	0	0	100%
		All	80	80	0	0	100%
*P. falciparum* – 2,000 parasites/μL	ID6, ID11, ID13, ID16	Health centre staff	20	0	20	0	100%
		Pharmacist	20	0	20	0	100%
		Village malaria worker	20	0	20	0	100%
		Physician	20	0	20	0	100%
		All	80	0	80	0	100%
*P. falciparum* - 200 parasites/μL	ID5, ID9, ID12, ID20	Health centre staff	20	0	20	0	100%
		Pharmacist	20	0	20	0	100%
		Village malaria worker	20	0	20	0	100%
		Physician	20	0	20	0	100%
		All	80	0	80	0	100%
*P. vivax* – 2,000 parasites/μL	ID1, ID7, ID10, ID14	Health centre staff	20	0	0	20	100%
		Pharmacist	20	0	0	20	100%
		Village malaria worker	20	0	0	20	100%
		Physician	20	0	0	20	100%
		All	80	0	0	80	100%
*P. vivax* - 200 parasites/μL	ID2	Health centre staff	5	5	0	0	0%
		Pharmacist	5	4	0	1	20%
		Village malaria worker	5	1	0	4	80%
		Physician	5	3	0	2	40%
		All	20	13	0	7	35%

No significant difference was observed between the different end-users. For the low positive (200 parasites/μL) *P. vivax* aliquot, the proportion of positive results obtained by the VMW end-users (80%) was higher than other end-users (0-40%), but the difference was not significant (P = 0.22), and was probably due to the low number of aliquots tests (five per end-user).

### Questionnaire evaluation analysis

After performing the test, all end-users were interviewed and completed a questionnaire (‘use and usability’) to evaluate ease-of-use and ease-of-reading of the VIKIA® Malaria test. Results are presented in Table 5. A total of 159 positive answers (yes, 99.4%) and only one negative answer (no, 0.6%) were recorded.

**Table 5:** **Results of the questionnaire evaluation, Kampot, Cambodia, 2013**

**Use of the test**	**No. of YES (%)**	**Comments**
Are the instructions given in the package insert easily understandable for use by you?	20/20 (100%)	
With only the support of the quick guide, is it easy to perform the test? If not, specify the part or parts which you do not easily understand:	19/20 (95%)	Part 3, I consider that one drop is equal to 5x
Is the test easy to use for someone who is not a trained laboratory technician? If not, please explain:	20/20 (100%)	
Are the operating instructions clear enough to understand the test without having to participate in practical training?	20/20 (100%)	
Is the time to perform the test compatible with its use in the field? If not, give an estimate of a time that seems to be acceptable, from sampling to result.	20/20 (100%)	1 comment: 15 min will be better
**Result interpretation**		
Do the instructions given in the package insert allow an easy interpretation of the results? If not, please explain:	20/20 (100%)	
With only the support of the quick guide, is the result easy to interpret?	20/20 (100%)	
Do you think that reading and result interpretation of the test are easy to read and interpret for someone who has not trained as a laboratory technician? If not, please explain:	20/20 (100%)	

Out of the 20 end-users, only one considered the test was not easy to perform with the support of the quick guide. However, with the help of the package insert, all end-users considered that the operating instructions were clear enough to perform the test without having to participate in practical training. Moreover, all end-users claimed that the package insert allowed an easy interpretation of the results.

## Discussion

In endemic areas, health facility staff and community workers are in the front line to manage malaria cases, which mostly affect rural inhabitants, for achieving the laudable goal of elimination. To this end, early diagnosis of malaria by using malaria RDT and prompt treatment with effective anti-malarial drugs are the cornerstone of the management of suspect malaria cases [11]. Therefore, malaria RDTs are one of the most important tools to ensure that only malaria-infected patients receive anti-malarial drugs, limiting the unnecessary use of inappropriate treatment and thereby avoiding selection and spread of drug-resistant *P. falciparum* parasites, an important issue in Southeast Asia [12]. According to WHO, most of the 108 million malaria RDTs delivered in 2012 were used in the African region (78%), followed by the Southeast Asia region (16%) and Eastern Mediterranean region (3%) [11].

In this context, the availability of accurate, high performance malaria RDTs in terms of sensitivity, specificity, positive, and negative predictive values is crucial. However, most of the studies aiming to evaluate the performance of commercialized malaria RDTs are conducted by research teams, that are not representative of the traditional end-users in remote areas, whereas several studies have clearly shown that the accuracy of malaria RDTs is highly user-dependent [13-20]. Indeed, it has been demonstrated by Rennie *et al.* that despite their apparent simplicity of use, the accuracy of malaria RDTs also depends on the accuracy of their preparation and interpretation [21]. In most of the studies, data relating to the quality of packaging and content, ease of use and ease of interpretation of results from malaria RDTs are missing [22].

In the present study, a dedicated protocol was designed to re-evaluate the performance of the VIKIA® Malaria Ag Pf/Pan test, previously assessed in 2013 [9], by representative end-users. To this end, well-characterized and calibrated blood samples were used, along with in-depth interviews to assess ease-of-use and ease-of-reading of the VIKIA® Malaria Ag Pf/Pan test. Globally, the performance of the VIKIA® Malaria Ag Pf/Pan test performed by the 20 selected end-users was consistent with expected results and similar to those previously observed by Chou *et al.* [9]. No significant difference was observed between the different end-users, including health medical centre staff, private physicians, community health workers and private pharmacists. With the instructions provided in the quick guide and the package insert, most of the end-users considered that the VIKIA® Malaria Ag Pf/Pan test was easy to use and the results were easy to interpret.

The results observed with the low positive *P. vivax* aliquot (200 parasites/μL) with a sensitivity of 35% (95% CI: 15.4-59.2%) were similar to the sensitivity reported for *P. vivax* samples containing 101–500 parasitaemia/μL of blood by Chou *et al.* (from 36.4 to 61.9%, according to the reading time, 10–60 minutes) [9]. In addition, data from the quality control assessment performed at three different times (at the start, middle and end of the study), confirmed that the storage of low parasitaemia *P. vivax* samples at low temperatures had probably accelerated the deterioration of antigen activity (among the 12 low positive *P. vivax* aliquots, seven were positive and five were negative) [7]. The 200 parasites/μL density level was included in the protocol according to the 1999 and 2003 WHO consultations (above the 100 parasites/μL level) [23,24]. The 2010 WHO consultation on parasite detection confirmed the relevance of this level for clinical management, in high and medium/low transmission areas [25], taking into account that clinical malaria at low parasite densities occurs in a number of communities, especially in unstable low transmission areas.

The main limitations of the study were the limited number of samples tested, especially the number of low positive *P. vivax* aliquots and the use of frozen samples, although no other option was available. Indeed, it has been claimed by some RDT manufacturers that frozen blood samples are not optimum for RDT testing. They argue that malaria RDTs are optimized and recommended to be used with fresh finger-prick blood and that frozen blood samples could lead to aggregation, precipitation and leeching out of artefacts into the thawed blood. As all RDTs use a porous nitrocellulose membrane, the presence of artefacts clogging the membrane can alter the flow properties of the reagents and affect the completion of the test run in specified time. As a future option, WHO, TDR and FIND, along with other partners, have started the development of a generation of lot-testing samples based on the use of recombinant proteins, which could be used by community health workers to confirm quality of RDTs and significantly increase the confidence of national malaria control programmes in the results of RDTs.

## Conclusions

The data presented in this study clearly demonstrate that the performance of the VIKIA® Malaria AgPf/Pan rapid test performed by traditional end-users in field conditions is similar to those obtained by a research team, and that VIKIA® Malaria AgPf/Pan can be considered as a point-of-care test. In addition, the protocol designed for this study, using well-characterized and calibrated blood samples at 2,000 and 200 parasites/μL could be used systematically in parallel to conventional evaluation studies [7] to determine the performance of malaria RDTs in field conditions.

## Additional files

Additional file 1:
**Quick guide for using the VIKIA® Malaria Ag Pf/Pan RDT.**
Additional file 2:
**Package Insert of the VIKIA® Malaria Ag Pf/Pan RDT.**
