# Supplementary figures and images for: Malaria rapid diagnostic test as point-of-care test: study protocol for evaluating the VIKIA® Malaria Ag Pf/Pan
Source: Malar J. 2015 Mar 14;14:114. doi: 10.1186/s12936-015-0633-3 (PMC4389415; doi:10.1186/s12936-015-0633-3)

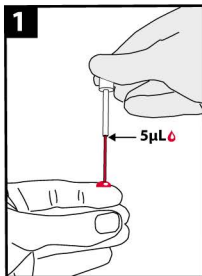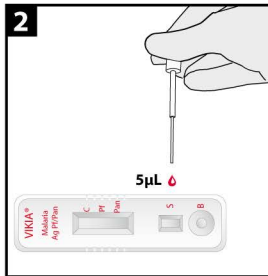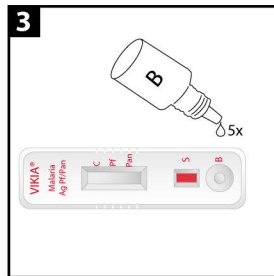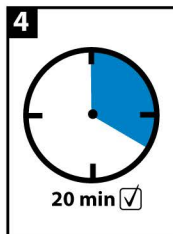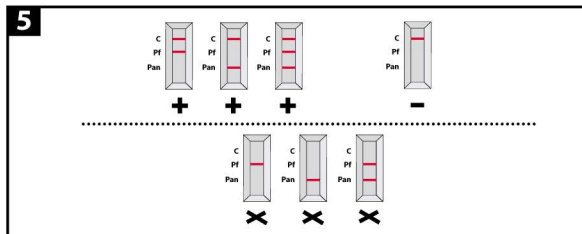

Supplement: Additional file 1: — Quick guide for using the VIKIA® Malaria Ag Pf/Pan RDT. [file 12936_2015_633_MOESM1_ESM.pdf]
