# Supplementary material for: Malaria rapid diagnostic test as point-of-care test: study protocol for evaluating the VIKIA® Malaria Ag Pf/Pan
Source: Malar J. 2015 Mar 14;14:114. doi: 10.1186/s12936-015-0633-3 (PMC4389415; doi:10.1186/s12936-015-0633-3)
Supplement: Additional file 2: — Package Insert of the VIKIA® Malaria Ag Pf/Pan RDT. [file 12936_2015_633_MOESM2_ESM.pdf]

VIKIA® Malaria Ag Pf/Pan

IVD

Rapid test for the qualitative detection of Plasmodium antigen in human blood specimens (venous blood or capillary blood). This kit is intended to be used as an aid in the diagnosis of malaria infections and the differential diagnosis of Plasmodium falciparum from other malarial infections. The test is for professional use only.

SUMMARY AND EXPLANATION

Malaria is one of the world's most prevalent diseases. According to the WHO, the worldwide prevalence of the disease is estimated to be 216 million cases, with over 655,000 deaths in 2010, 86% of which are children under the age of 5 years (1). Over half of the world's population lives in malarious areas.
Five species of Plasmodium parasites are responsible for the infection: *Plasmodium falciparum (P.f.)*, *Plasmodium vivax* (2) *(P.v.)*, *Plasmodium ovale (P.o.)*, *Plasmodium malariae (P.m.)* and *Plasmodium knowlesi* (3). Differentiation of the Pf species from other species is very important due to its higher mortality and morbidity (4), as well as its resistance to antimalarial treatment (5). Rapid and effective diagnosis of the disease is therefore necessary and the VIKIA® Malaria Ag Pf/Pan test meets these requirements.

PRINCIPLE

The VIKIA® Malaria Ag Pf/Pan rapid test is an immunochromatographic test in which monoclonal antibodies target:

- the HRP-II protein, antigen specific to *Plasmodium falciparum (P.f.)* (6)
- the Pan (Aldolase) antigen, common to all the Plasmodium species (7)

The blood specimen is dispensed in the sample well, followed by the addition of the erythrocyte lysis buffer in the buffer well, which releases the antigens. Then the migration occurs by capillary flow on the membrane. If the sample contains antigens, they will make complexes with specific antibodies coupled to gold particles. These complexes will migrate along the membrane up to the Pf and/or Pan regions where they are captured by immobilized antibodies on the membrane, resulting in the formation of one or two red lines. The presence of a red internal control band validates the proper functioning of the test.

CONTENT OF THE KIT (25 TESTS):

25 sealed pouches		Cassettes + Plastic capillary pipettes + Desiccant
1 vial of running lysis buffer	B	Allows lysis of erythrocytes and migration
1 quick guide, printed on the box		
1 Package insert		

MATERIAL REQUIRED BUT NOT PROVIDED

- Timer
- Equipment for taking blood samples by venipuncture or finger stick

WARNINGS AND PRECAUTIONS

- **For *in vitro* diagnosis only.**
- **For professional use only.**
- This kit contains products of animal origin. Certified knowledge of the origin and/or sanitary state of the animals does not totally guarantee the absence of transmissible pathogenic agents. It is therefore recommended that these products be treated as potentially infectious, and handled observing the usual safety precautions (do not ingest or inhale).
- Do not use reagents after the expiry date indicated on the packaging.
- Do not touch the test device chromatography membrane with your fingers.
- During the test, the device must be placed on a flat vibration-free surface. Do not shake the device while running the test.
- The test device should be stored in the sealed pouch containing the desiccant until use.
- The test device and the capillary pipette are single use only: they should not be reused.
- Do not mix reagents from different batches.
- As the specimens are potentially infectious, wear gloves when handling them.
- The running buffer contains low amounts of sodium azide (preservative).
- This package insert should be read completely before performing the test. Failure to follow the insert may give inaccurate test results.
- The test is designed to be used with human blood only. All clinical specimens should be considered potentially infectious and handled following the recommended precautions (see recommended country regulation). Wear protective clothing such as laboratory coats, disposable gloves and eye protection when specimens are assayed
- Discard used or unused reagents as well as any other contaminated disposable materials following procedures for infectious or potentially infectious products.

STORAGE CONDITIONS AND STABILITY

- Store the kit at 2-30°C.
- **DO NOT FREEZE.**
- If stored under the recommended conditions, all components are stable until the expiry date indicated on the packaging. Do not use after the expiry date.
- The test device should remain in the sealed pouch until use.

SPECIMEN COLLECTION AND PREPARATION

Preparation

Before use, the reagents must be brought to room temperature (15-30°C).
Have the cassette, the pipette, the buffer and the timer ready for use if blood from a finger prick is used to avoid clotting of the blood sample.

Specimen collection

Collection of whole blood by venipuncture

1. Collect blood specimen in an appropriate collection tube (containing EDTA, oxalate, citrate or heparin as an anticoagulant) by venipuncture.
2. Take the provided plastic capillary pipette (see image below) and aspirate blood by filling only the fine tip of the pipette (5µL).
3. If immediate testing is not possible blood samples can be stored refrigerated between 2-8°C up to 48 hours.

Collection of capillary blood from finger prick

1. Clean the puncture site with antiseptic and allow to air dry.
2. Massage the hand towards the fingertips without touching the puncture site.
3. Prick the fingertip with a lancet. Wipe the first drop of blood.
4. Take the provided plastic capillary pipette and aspirate blood by filling only the fine tip of the pipette (5µL).
5. Start assay procedure immediately to avoid clotting of the blood.

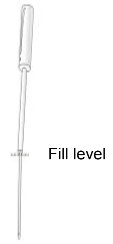

Capillary pipette

INSTRUCTIONS FOR USE

Allow the required reagents to come to room temperature before use.

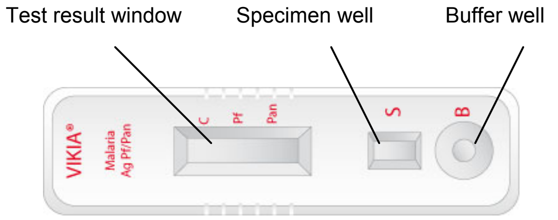

1. Transfer the sample completely into the specimen well S.
2. After opening, hold the opened buffer vial vertically and dispense 5 drops of buffer into the buffer well "B". Avoid trapping air bubbles in the buffer well and do not drop any solution in the test result window or the specimen well.
3. The result should be read after 20 minutes in the test result window. Do not interpret results after more than 30 minutes.

NB: Occasionally the background of the test will not clear completely but will still show a faint reddish color due to the hemoglobin from the lysed erythrocytes. This does not interfere with the interpretation of the result as long as the lines are not obscured.

RESULTS AND INTERPRETATION

The reading and interpretation of the test is done based on the lines that appear in the test result window:

Negative result

Only one red colored line appears in the control region (C). No apparent colored lines appear in the Pf and the Pan test regions. This result indicates that no malarial antigen has been detected by the assay.

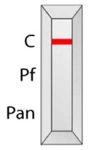

Positive result for *P. falciparum*

Two red colored lines appear on the membrane. One line appears in the control region (C) and another line appears in the Pf test region. This result indicates a *P. falciparum* infection.

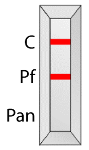

Positive result for *P. vivax, P. ovale* and/or *P. malariae*

Two red colored lines appear on the membrane. One line appears in the control region (C) and another line appears in the Pan test region. This result indicates an infection with *P. vivax*, *P. ovale* or *P. malariae* alone or a mixed infection with these species. No *P. falciparum* antigen has been detected with the assay.

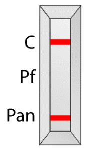

Positive result for *P. falciparum* or mixed infection

Three red colored lines appear on the membrane. One line appears in the control region (C) and the other two lines appear in the Pf and Pan test regions. This result indicates a *P. falciparum* infection or a mixed infection with *P. falciparum* and at least one other Plasmodium species (*P. vivax*, *P. ovale* and/or *P. malariae*).

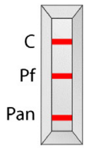

Invalid Result

The control band fails to appear. In this case, the results should be discarded. Read the instructions again and repeat the test with a new device.

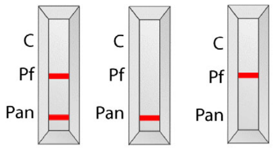

NOTES:

THE TEST IS QUALITATIVE ONLY. THE INTENSITY OF THE RED COLOR IN THE PF AND PAN TEST REGIONS MAY VARY.

QUALITY CONTROL

The test includes an internal control of migration indicated by a red colored line in the control region (C). If this control line does not appear, the test is invalid.

NB: It is the responsibility of the user to perform the Quality Control in accordance with local applicable regulations.

LIMITATIONS OF THE TEST

1. The VIKIA® Malaria Ag Pf/Pan Rapid Test Device is for professional use only, and should be used for the qualitative detection of malaria antigens only.
2. If the test result is negative and clinical symptoms persist, additional testing using other biological methods (e.g. microscopic examination of the thick smear and thin blood films) is recommended. A negative result does not at anytime rule out the existence of malaria antigens in blood, because the antigens may be below the minimum detection level of the test.
3. The diagnosis should be confirmed by a physician after all clinical and laboratory findings have been evaluated.
4. Specimens with positive titers for rheumatoid factors may sometimes give positive results.
5. Specimens with positive titers for human anti-mouse antibodies (HAMA) may sometimes give positive results.

PERFORMANCE

The performance characteristics of the VIKIA® Malaria Ag Pf/Pan test were established during a multicenter study conducted in Southeast Asia and sub-Saharan Africa. The blood specimens were obtained from patients with malaria-like symptoms. The test was done through comparison with microscopic examination (thick smear and thin blood films). Real-time PCR was done in order to determine the discordant cases and confirm the species diagnosis [8].

The clinical studies performed with VIKIA® Malaria Ag Pf/Pan showed the following results:

Sensitivity compared to microscopy

	**Total (Southeast Asia & sub-Saharan Africa)**	
	**% Sensitivity**	**CI (95%)**
P. falciparum & Mixed infections *P.f.* + other species of Plasmodium	**96.3%** (283/294)	[93.4 - 97.9%]
P. vivax, *P. ovale* or *P. malariae*	**89.1%** (197/221)	[84.2 - 92.7%]

Sensitivity compared to parasite density*

	***P. falciparum* (Pf)**		**Non *P. falciparum* (Pan)**	
**Parasitemia/µL of blood**	**% Sensitivity**	**CI (95%)**	**% Sensitivity**	**CI (95%)**
**≥ 200**	**97.3%** (183/188)	[93.8 - 98.9%]	**91.8%** (179/195)	[87 - 94.9%]
**≥ 2000**	**100%** (100/100)	na	**97%** (162/167)	[93.1 - 98.7%]

* mixed infections (*Pf* + other species of Plasmodium) were not taken into consideration

Specificity

Determined with specimens confirmed negative by microscopy and real-time PCR.

**Total (Southeast Asia & sub-Saharan Africa)**	
**% Specificity**	**CI (95%)**
**98%** (1145/1168)	[97 - 98.7%]

WASTE DISPOSAL

Discard used or unused reagents as well as
any other contaminated disposable materials following procedures for infectious or potentially infectious products.
It is the responsibility of each laboratory to handle waste and effluents produced according to their nature and degree of hazardousness and to treat and dispose of them (or have them treated and disposed of) in accordance with any applicable regulations.

ASSISTANCE

If you have any questions concerning the use of the product, contact the local bioMérieux representative or bioMérieux (www.biomerieux.com).

LITERATURE REFERENCES

1. World Health Organization. World malaria report 2011. Geneva, Switzerland: WHO Press; 2011
2. Price RN, Tjitra E, Guerra CA, Yeung S, White NJ, et al. (2007) Vivax malaria: neglected and not benign. Am J Trop Med Hyg 77: 79–87
3. Cox-Singh J, et al. *Plasmodium knowlesi* malaria in humans is widely distributed and potentially life-threatening. Clin. Infect. Dis. 2008;46:165–171
4. SNOW, R. W., GUERRA, C. A., NOOR, A. M., MYINT, H. Y. & HAY, S. I. (2005). The global distribution of clinical episodes of *Plasmodium falciparum* malaria. Nature 434, 214–217
5. Korenromp E, Miller J, Nahlen B, Wardlaw T, Young M. World Malaria Report 2005. World Health Organization, Roll Back Malaria, Department and the United Nations Children's Fund. 2005
6. Baker J, McCarthy J, Gatton M, Kyle DE, Belizario V, Luchavez J, et al. Genetic diversity of *Plasmodium falciparum* histidine-rich protein 2 (PfHRP2) and its effect on the performance of PfHRP2-based rapid diagnostic tests. J Infect Dis 2005;192:870-7
7. Lee, N., J. Baker, D. Bell, J. McCarthy, and Q. Cheng. 2006. Assessing the genetic diversity of the aldolase genes of *Plasmodium falciparum* and *Plasmodium vivax* and its potential effect on performance of aldolase-detecting rapid diagnostic tests. J. Clin. Microbiol. 44:4547-4549.
8. Maeno Y,Nakazawa S, Dao le D, et al. A dried blood sample on filter paper is suitable for detecting Plasmodium falciparum gametocytes by reverse transcription polymerase chain reaction. Acta Trop 2008;107:121-7.

INDEX OF SYMBOLS

Symbol	Meaning
REF	Catalog number
IVD	*In vitro* diagnostic medical device
	Manufacturer
	Temperature limitation
	Use by
LOT	Batch code
	Consult Instructions for Use
Σ	Contents sufficient for "n" tests
	Do not reuse

Distributed by: **bioMérieux® SA**
Chemin de l'Orme
69280 Marcy-l'Etoile - France
RCS LYON 673 620 399
Tel. 33 (0)4 78 87 20 00
Fax 33 (0)4 78 87 20 90
www.biomerieux.com

BIOMERIEUX, the blue logo and VIKIA are used, pending and/or registered trademarks belonging to bioMérieux or one of its subsidiaries, or one of its companies.
Any other name or trademark is the property of its respective owner.

IMACCESS

IMACCESS SAS
321, avenue Jean Jaurès
Bâtiment Domilyon – Centre d'Infectiologie
69007 Lyon / France

CE
